# Drug-coated balloons in coronary in-stent restenosis: a systematic review and meta-analysis comparing sirolimus and biolimus with paclitaxel platforms

**DOI:** 10.1007/s12928-026-01248-4

**Published:** 2026-03-03

**Authors:** Mrunalini Dandamudi, Muhammad Aqib Faizan, Tooba Rehman, Caterina Lecchi, Manoj Ghimire, Jyothik Varun Inampudi, John Cedric Mojica, Salomon Chamay, Juliana Giorgi

**Affiliations:** 1 Montefiore Einstein Medical Center, Bronx, NY USA; 2 Gomal Medical College, Khyber Medical University, Peshawar, Pakistan; 3 Università Della Svizzera Italiana, Lugano, Switzerland; 4 Barnabas Health System, Bronx, NY USA; 5 Detroit Medical Center, Wayne State University, Detroit, USA; 6 University of the East Ramon Magsaysay Memorial Medical Center, Quezon City, Philippines; 7 Morristown Medical Center, Morristown, New Jersey USA; 8 Hospital Sirio Libanes, São Paulo, Brazil; 9 Hospital Albert Einstein, São Paulo, Brazil

**Keywords:** Coronary in-stent restenosis, Drug coated balloons, Sirolimus, Biolimus, Paclitaxel, Target lesion revascularization, Drug elluting stent, Bare metal stent, Late lumen loss, Target vessel MI

## Abstract

**Background** Coronary in-stent restenosis (ISR) remains a relevant clinical challenge despite advances in drug-eluting stent technology. Drug-coated balloons (DCBs) offer a metal-free revascularization option, with microtubule stabilizer drug – paclitaxel coated balloons (PCBs) being the most validated. The m-TOR inhibitors drugs like Sirolimus and Biolimus coated balloons (SCBs, BCBs) have emerged as alternatives, but comparative efficacy and safety remain uncertain. **Objective** To compare the efficacy and safety of SCBs and BCBs with PCBs in the treatment of coronary ISR. **Methods** A systematic review and meta-analysis of randomized controlled trials (RCTs) was conducted according to PRISMA guidelines. Multiple databases were searched through February 2025 for RCTs comparing SCB or BCB with PCB in ISR. The primary outcome was target lesion revascularization (TLR). Secondary outcomes included mortality, target vessel myocardial infarction (TVMI), binary restenosis, late lumen loss (LLL), percentage diameter stenosis, and minimal lumen diameter (MLD). **Results** Six RCTs involving total of 988 patients with coronary ISR (due to DES n = 887 and BMS n = 92) were analyzed. SCB/BCB use was associated with a non-significant trend toward higher TLR (RR = 1.24, 95% CI 0.91–1.69) compared with PCB. No significant differences were observed for mortality, TVMI, binary restenosis, LLL, diameter stenosis, or MLD, even upon subgroup analysis based on type of drug coated balloon used and etiology of ISR (DES-ISR or DES + BMS-ISR). **Conclusion** Our study finds that SCBs and BCBs are non-inferior to PCBs in treatment of coronary ISR in terms of safety and efficacy, though criteria for selection of type of balloon to be utilized could not be derived as studies included had comparable baseline characteristics and subgroup analysis did not reveal any difference in results as well. However, given the limited number of direct comparisons, low certainty of evidence for many outcomes, and a numerically higher trend for TLR in the SCB/BCB group, a definitive conclusion of equivalence cannot be drawn, and further research is warranted.

**Graphical Abstract:**

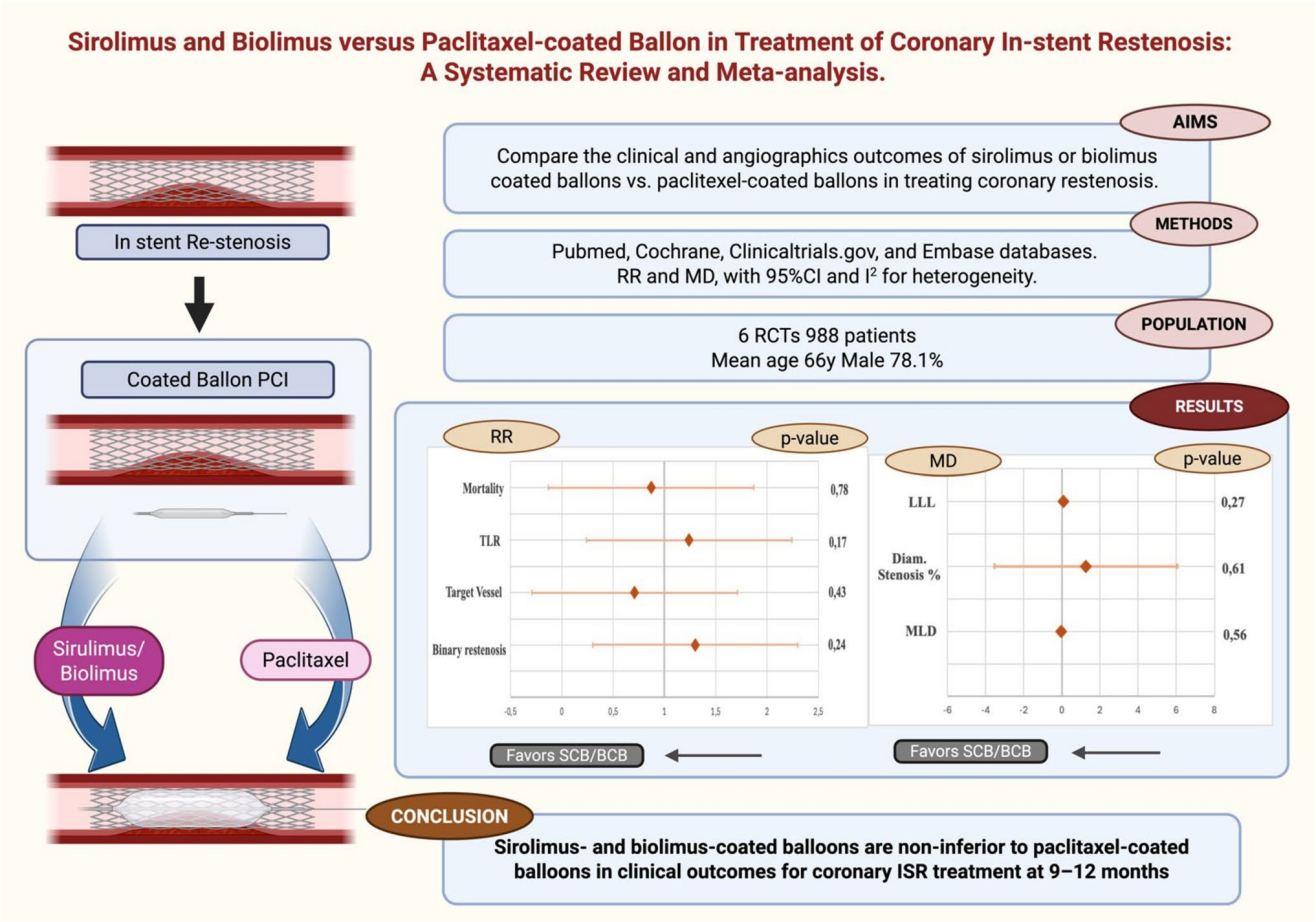

**Supplementary Information:**

The online version contains supplementary material available at 10.1007/s12928-026-01248-4.

## Introduction

Primary percutaneous coronary intervention (PCI) remains the preferred reperfusion method for acute coronary syndromes (ACS), with the latest-generation drug-eluting stents (DES) recommended for all patient and lesion types [1]. In-stent restenosis (ISR) continues to be the most common cause of PCI failure [2]. Drug-coated balloons (DCBs) have emerged as an effective alternative in selected cases, especially for patients with ISR, high bleeding risk, or small vessel coronary artery disease (CAD) [1]. By avoiding permanent implants and vessel caging, DCBs permit shorter courses of dual antiplatelet therapy and, over the medium to long term, can promote late lumen enlargement (LLE), vessel remodeling, and plaque regression [3]. Since 2014, the European Society of Cardiology has given DCBs a Class 1A recommendation for managing ISR [4].

The microtubule-stablizer drug (Paclitaxel) coated balloons (PCB) are currently the standard of care for treating coronary ISR [1]. Paclitaxel, a cytotoxic agent, binds irreversibly to beta-tubulin, preventing microtubule disassembly, which stabilizes microtubules, disrupts their function, and halts cell division [5]. Although m-TOR inhibitor drugs like sirolimus offers comparable safety, efficacy, and stronger anti-restenotic and anti-inflammatory properties than paclitaxel when delivered via a coronary stent, its use in DCBs presents challenges. Because DCBs require rapid drug release and short transfer times, sirolimus’s low lipophilicity limits its vessel wall penetration and retention [5]. Evidence from randomized controlled trials (RCTs) on m-TOR inhibitor drug coated balloons (like SCBs and BCBs) with direct comparisons between SCBs or BCBs and paclitaxel-coated balloons (PCBs) remain scarce. This meta-analysis aims to evaluate and compare the safety and efficacy profiles of SCBs and BCBs with PCBs.

## Methods

This systematic review and meta-analysis were conducted and reported in accordance with the Cochrane Collaboration Handbook for Systematic Reviews of Interventions and the Preferred Reporting Items for Systematic Reviews and Meta-Analysis (PRISMA) Statement guidelines [6]. The protocol was registered in the International Prospective Register of Systematic Reviews (PROSPERO) under record number CRD420251047870.

### Data source and search strategy

We systematically searched PubMed, Cochrane, Clinicaltrials.gov, and Embase databases for studies published from database inception to February 2025. The entire search strategy is mentioned in *Supplemental *Table 1. Two authors (M.D. and J.I.) independently evaluated full-text publications for inclusion based on predetermined criteria after eliminating duplicates and screening titles and abstracts. A panel discussion with the third author (M.G.) settled the disagreements.

**Table 1:** Baseline characteristics of included studies

Study	Age^†^	Type of ISR	Type of DCB	Male n (%)	BMI (kg/m^2^)^†^	DM n (%)	HTN n (%)	Previous MI n (%)	Previous/ current smoker, n (%)	F/U Duration
**Byrne 2025**	68.7 ± 10.0	BMS & DES	BCB Vs PCB	202 (80.2)	28.2 ± 4.5	59 (29.2)	169 (83.7)	106 (52.3)	32 (15.8)	12 months
**Liu** **2025**	63.8 ± 8.9	DES	SCB Vs PCB	192 (74.4)	N/A	97 (37.6)	177 (68.6)	14 (5.4)	N/A	12 months
**Pleva 2025**	68.8 ± 10.1	BMS & DES	SCB Vs PCB	115 (79.3)	29.0 ± 13.3	58 (40.0)	N/A	98 (67.6)	72 (49.7)	12 months
**Chen 2024**	63.9 ± 8.9	DES	BCB Vs PCB	204 (74.2)	25.9 ± 3.45	110 (40.0)	184 (66.9)	55 (20.0)	N/A	9 months
**Scheller 2022**	65.0 ± 12.1	DES	SCB Vs PCB	82 (81.2)	28.7 ± 4.8	57 (56.4)	97 (96.0)	56 (55.4)	17 (16.8)	12 months
**Scheller 2019**	60.1 ± 12.1	DES	SCB Vs PCB	41 (82.0)	N/A	37 (74.0)	47 (94.0)	17 (34.0)	6 (12.0)	12 months

^**†**^ Mean or Median, Study = Last name of Author and Year of publication

DM: Diabetes Mellitus, HTN: Hypertension, F/U: Follow up, DES: Drug eluting stent, BMS: Bare metal stent, ISR: In-stent restenosis, BMI: Body Mass Index; MI: Myocardial Infarction; kg/m^2^: kilogram per square meter

**Fig. 1:**
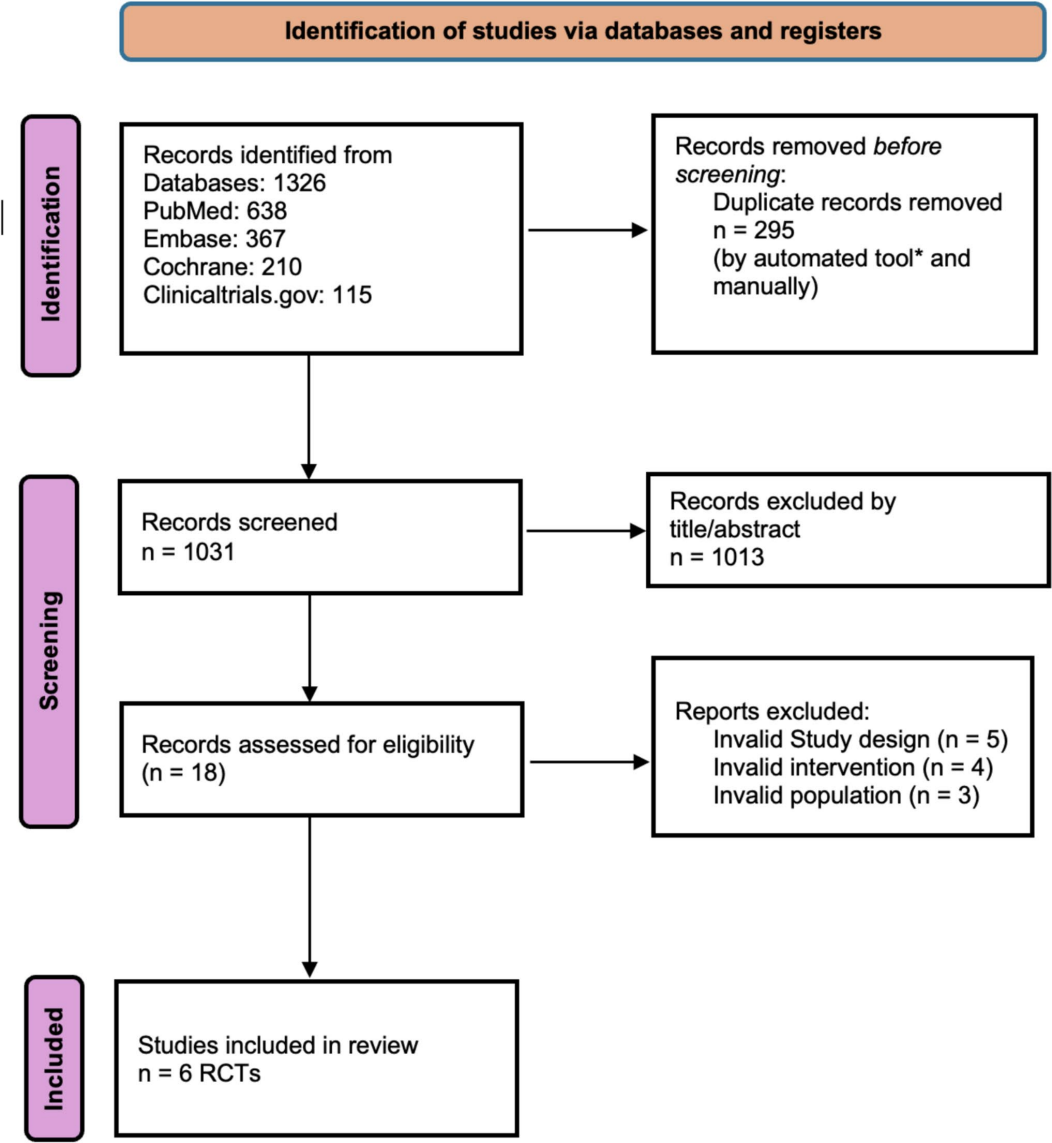
Prisma figure showing study selection. *Utilized the Rayyan automated screening tool to exclude records with exact title/author/DOI, as well as manually by two authors (M.D.) and (J.I)

Moreover, we searched for additional eligible studies by reviewing the references from the included studies. The PRISMA flowchart, illustrating the search results, can be found in Fig. 1*.*

### Eligibility criteria

Studies that fulfilled the following eligibility criteria were included: [1] Randomized control studies; [2] enrolling patients with coronary In-stent restenosis; [3] trials comparing efficacy of SCB/BCB versus PCB; and [4] reporting at least one common endpoint of interest that had been predetermined.

Exclusion criteria were: [1] absence of a control group; [2] patients without coronary In-stent restenosis; [3] studies enrolling ≤ 10 patients; [4] conference abstracts or [5] studies with overlapping populations.

### Data extraction

Data extraction was done independently by two authors (M.G. and J.M.), and disagreements were settled in a group discussion with the third author (M.D.). Using a standard data extraction form, the first author of the studies, the publication year, sample size, patient characteristics (age, sex ratio), intervention details, and clinical outcomes were all included in the data extracted from the eligible studies. For categorical outcomes, we retrieved the total number of patients and the number of incidents with the Risk ratio for each outcome; for continuous outcomes, we recovered the sample size, mean, and standard deviations with a confidence interval. Baseline characteristics were reported as the mean ± standard deviation for continuous variables and Risk Ratio (RR) for binary variables.

### Study endpoints and subgroup analyses

Outcomes of interest included Target Lesion Revascularization (TLR), Mortality, Target Vessel Myocardial infarction, Binary restenosis, Late Lumen Loss (LLL), Diameter stenosis in percentage, and Minimal lumen diameter (MLD). We performed subgroup analysis based on individual use of SCB or BCB to determine the efficacy and safety of the intervention independently. Heterogeneity was assessed using I^2^ statistics, and a two-tailed *p*-value of < 0.05 indicates statistical significance.

### Statistical analysis

The statistical analyses and graphical outputs were generated using R software (version 4.3.1) with the “metafor” and “metabin” packages and calculated the Risk Ratios (RR) for dichotomous outcomes along with the corresponding 95% Confidence Intervals (CIs). A random-effects model Mantel–Haenszel method was employed to address heterogeneity among studies. The pooled results were represented graphically as forest plots. The restricted maximum likelihood estimator was used to calculate heterogeneity variance tau [2]. Heterogeneity was assessed with Cochrane’s Q statistic and Higgins and Thompson’s I^2^ statistic [7]. The consistency of the studies was determined based on I^2^ values of 0%, ≤ 25%, ≤ 50%, and > 50%, indicating no observed, low, moderate, and substantial heterogeneity, respectively. Statistical significance was indicated by *p*-values < 0.05.

### GRADE certainty assessment

The certainty of evidence for each outcome was evaluated using the GRADE (Grading of Recommendations Assessment, Development and Evaluation) framework. We employed the GRADEpro GDT software to assess evidence quality across five key domains. A Summary of Findings (SoF) table was generated to display relative and absolute effects, participant numbers, and the overall confidence in the estimates.

### Quality assessment

We assessed the quality of studies using the Cochrane RoB-2 tool [8]. Each study was assessed in five domains: randomization, deviation from intended intervention, missing outcome data, measurement of the outcomes, and selection of the reported results.

## Results

### Baseline characteristics

This meta-analysis included 6 randomized controlled trials [9–14] with a total of 988 patients with coronary In-stent restenosis, where 527 participants in the SCB(n = 252)/BCB group (n = 275) were compared with 458 patients in PCB group across various clinical endpoints. The mean age of the patients across the studies was 65.4 years, mean BMI of 27.54 kg/m^2^ and the follow up duration was 9–12 months. The detailed characteristics of the individual studies are provided in Table 1.

### Results

The pooled analysis demonstrated comparative results between SCB or BCB and PCB across various clinical efficacy and safety endpoints, suggesting no clear superiority of one treatment modality over the other.

**Fig. 2:**
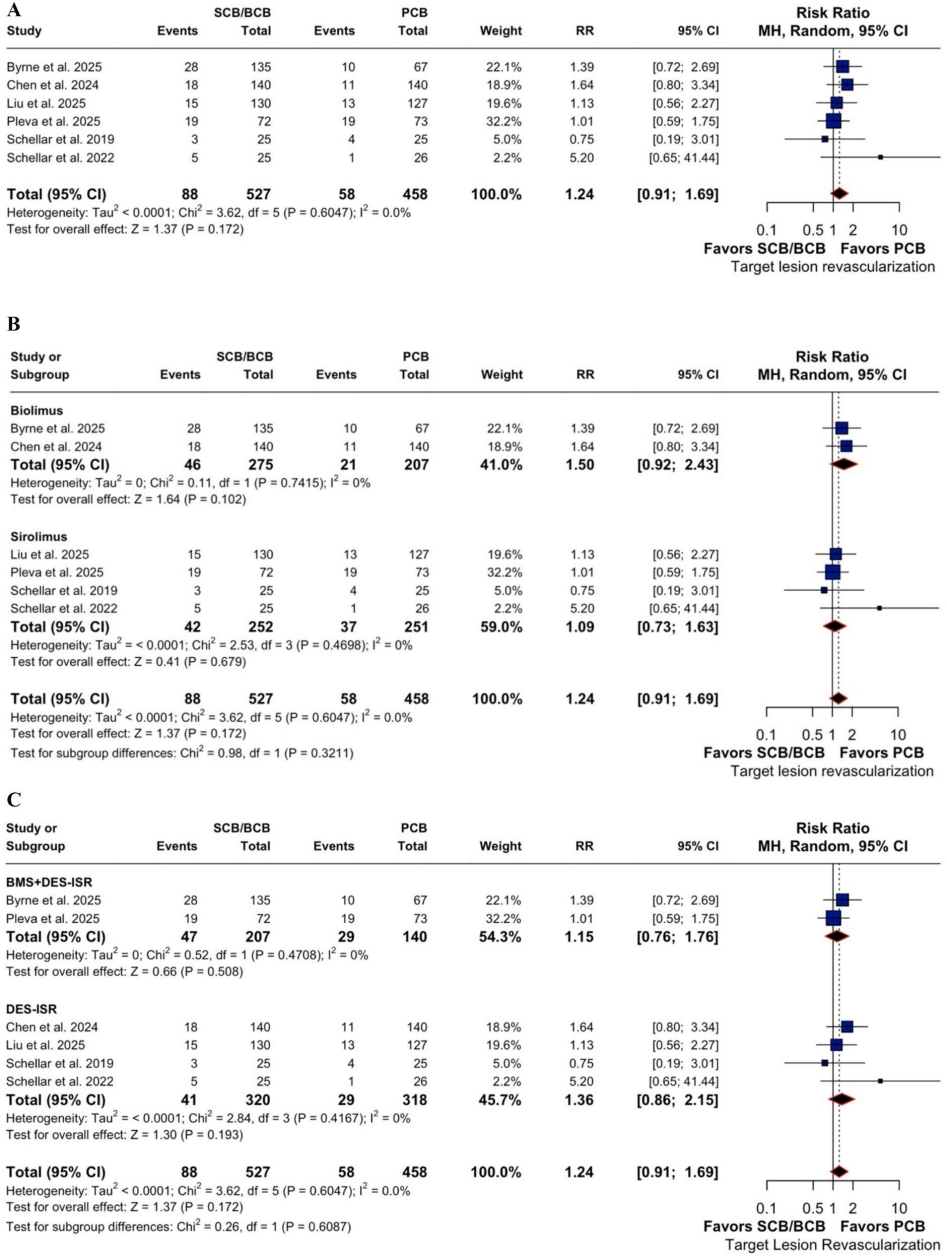
**A**: Pooled analysis of Target lesion revascularization (TLR) at follow-up comparing both SCB and BCB with PCB in Coronary ISR. **B**: Subgroup analysis based on type of balloon (SCB or BCB vs PCB) for TLR at follow-up. **C**: Subgroup analysis based on type of prior stent (DES/BMS) In-stent restenosis (ISR) for TLR at follow-up

The Target lesion revascularization (TLR) did not show any statistically significant difference between both the groups with null heterogeneity (RR = 1.24, 95%CI = 0.91, 1.69, p = 0.17, I^2^ = 0% Fig. 2A), and subgroup analysis comparing both SCB and BCB independently with PCB showed similar results with no subgroup difference (p = 0.32 Fig. 2B). Subgroup analysis based on type of ISR whether DES and BMS-ISR or DES-ISR, also showed similar results with test for subgroup showing no significance (p = 0.6 Fig. 2C).

**Fig. 3:**
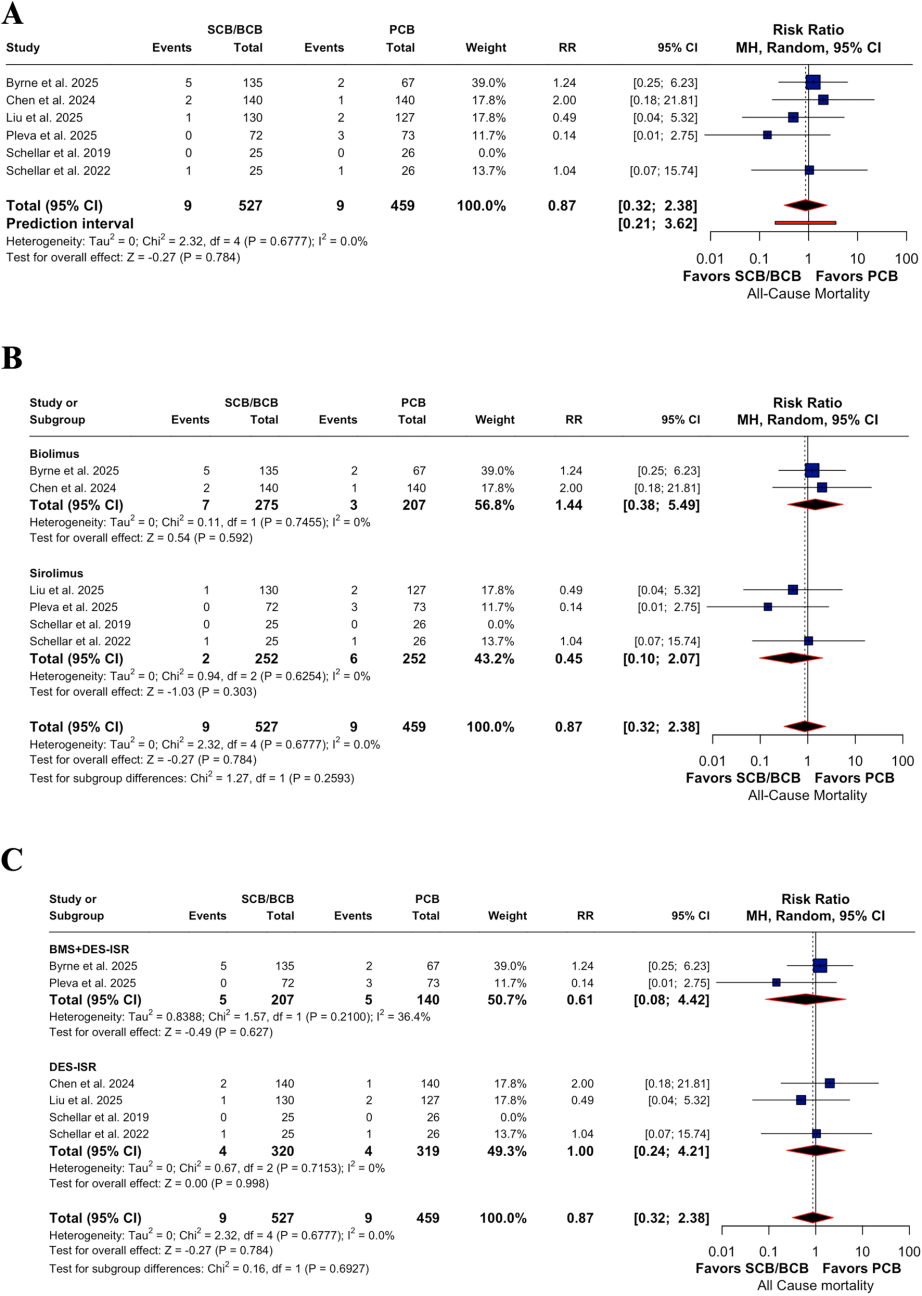
**A**: Pooled analysis of All-Cause mortality (ACM) at follow-up comparing both SCB and BCB with PCB in Coronary ISR. **B**: Subgroup analysis based on type of balloon (SCB or BCB vs PCB) for ACM at follow-up. **C**: Subgroup analysis based on type of prior stent (DES/BMS) In-stent restenosis (ISR) for ACM at follow-up

The all-cause mortality did not show any difference whether the patients were treated with SCB/BCB vs PCB (RR = 0.87, 95%CI = 0.32, 2.38, p = 0.78, I^2^ = 0% Fig. 3A), and subgroup analysis based on type of balloon utilized and type of ISR showed similar results with no significant subgroup difference (Fig. 3B, 3C).

**Fig. 4:**
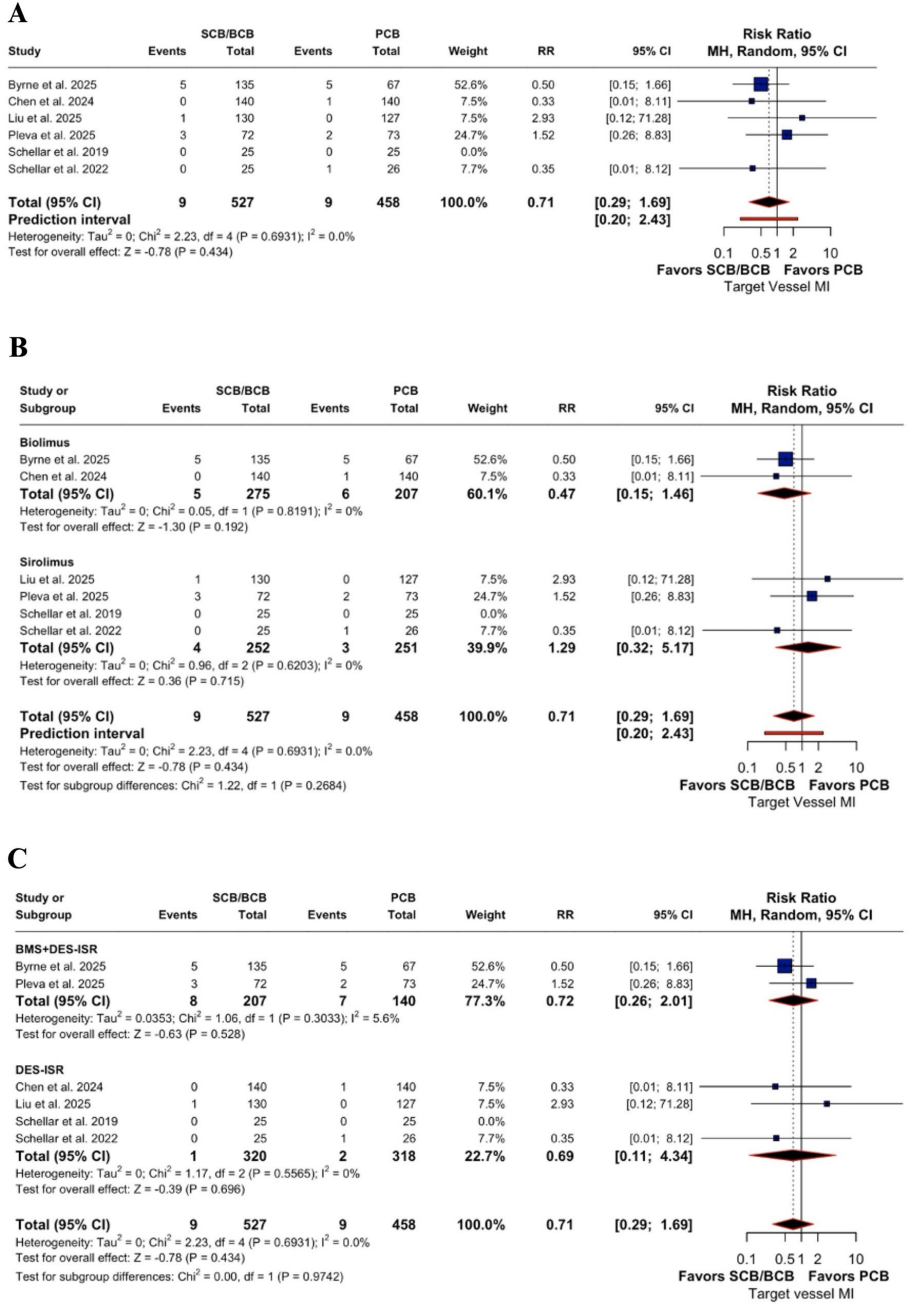
**A**: Pooled analysis of Target vessel Myocardial Infarction (TVMI) at follow-up comparing both SCB and BCB with PCB in Coronary ISR. **B**: Subgroup analysis based on type of balloon (SCB or BCB vs PCB) for TVMI at follow-up. **C**: Subgroup analysis based on type of prior stent (DES/BMS) In-stent restenosis (ISR) for TVMI at follow-up

**Fig. 5:**
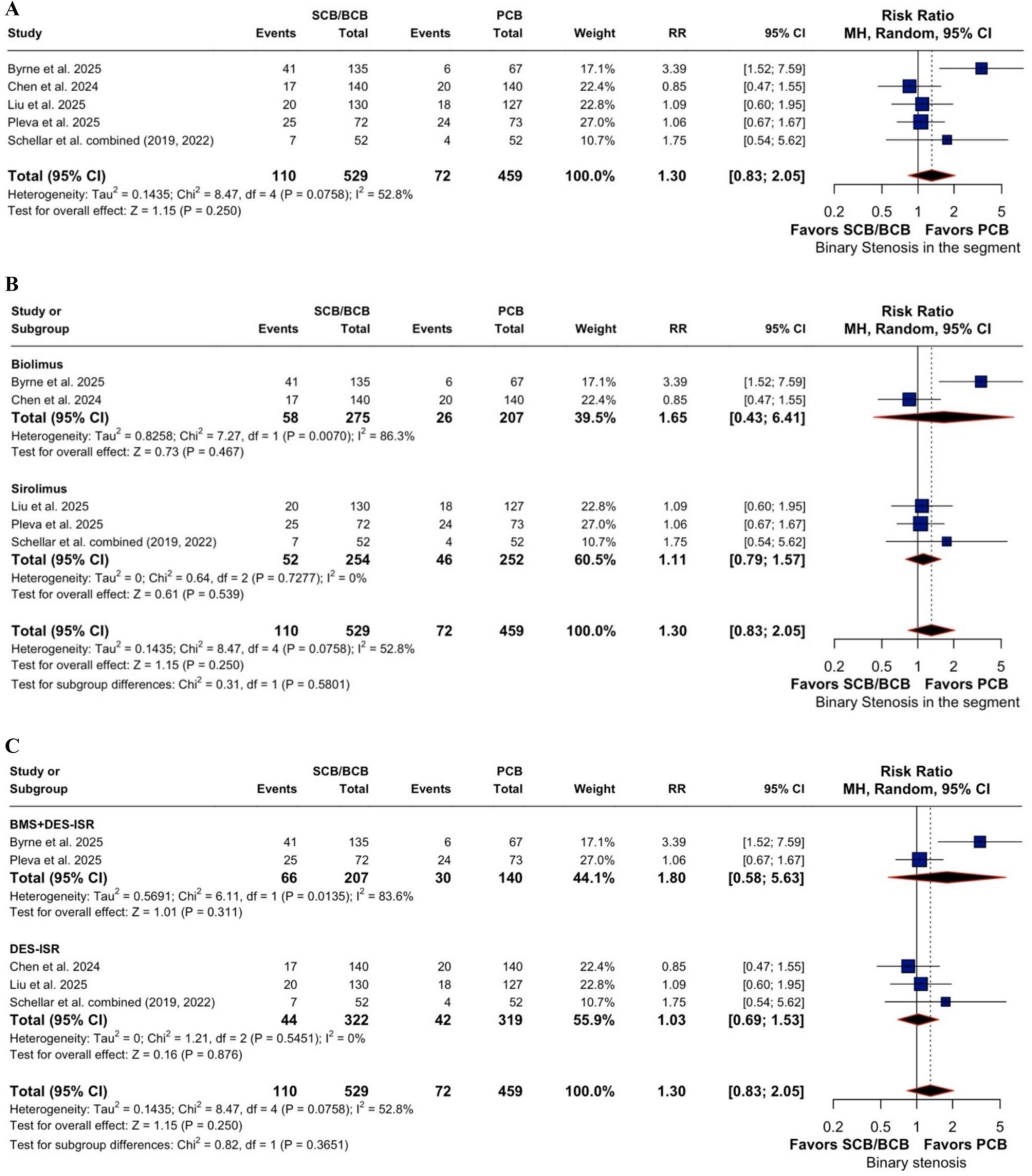
**A**: Pooled analysis Binary stenosis (BS) in segment at follow-up comparing both SCB and BCB with PCB in Coronary ISR. **B**: Subgroup analysis based on type of balloon (SCB or BCB vs PCB) for Binary stenosis at follow-up. **C**: Subgroup analysis based on type of prior stent (DES/BMS) In-stent restenosis (ISR) for BS at follow-up

A similar trend was noted in target vessel myocardial infarction (TVMI) (RR = 0.71, 95% CI = 0.29, 1.69, P = 0.43, I^2^ = 0% Fig. 4A, 4B, 4C) suggesting similar results upon pooled analysis and subgroup analysis. Binary restenosis percentage did not differ in patients treated with either SCB/BCB or PCB (RR = 1.30, 95% CI = 0.83, 2.05, P = 0.25, I^2^ = 53% Fig. 5A**),** despite subgroup analyses based on the type of balloon utilized, SCB vs PCB and BCB vs PCB (p = 0.58 Fig. 5B) and based on type of stent of ISR with test for subgroup statistically non-significant (p = 0.36 Fig. 5C).

**Fig. 6:**
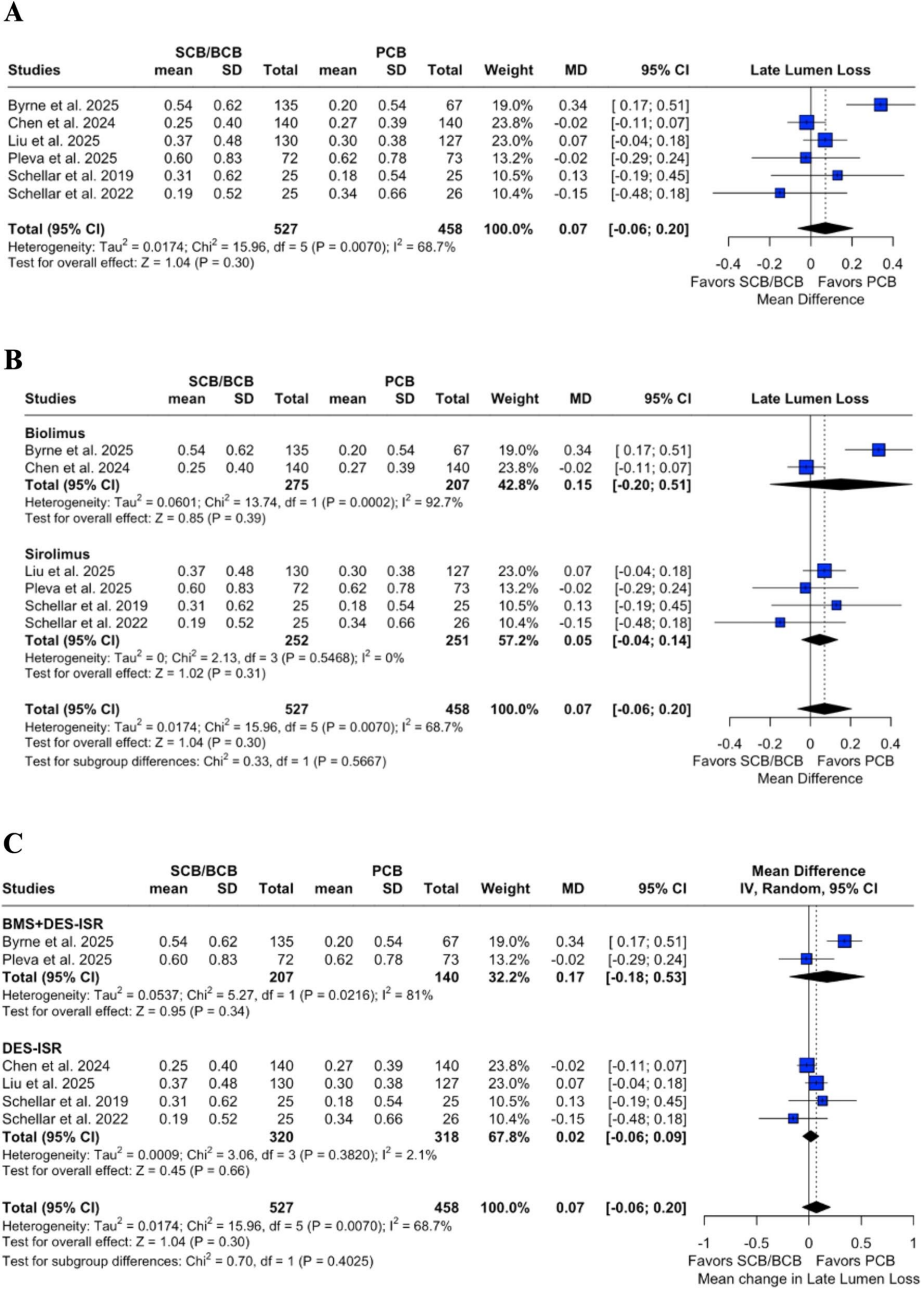
**A**: Pooled analysis of Late Lumen Loss (LLL) at follow-up comparing both SCB and BCB with PCB. **B**: Subgroup analysis based on type of balloon (SCB or BCB vs PCB) for LLL at follow-up. **C**: Subgroup analysis based on type of prior stent (DES/BMS) In-stent restenosis (ISR) for LLL at follow-up

Late lumen loss (LLL) suggests more tissue growth and higher likelihood of restenosis, mean difference (MD) of late lumen loss is 0.07 (95% CI = -0.06, 0.20, p = 0.30, I^2^ = 69% Fig. 6A) when compared between both the groups, and when SCB was compared with PCB (MD: 0.05 95% CI: -0.04, 0,14 p = 0.31, I^2^ = 0% Fig. 6B). When BCB was compared with PCB, late lumen loss shows a MD: 0.15 (95% CI -0.20, 0.51, p = 0.39 I^2^ = 93% Fig. 6B) with very high heterogeneity. Despite subgroup analysis based on type of ISR (DES + BMS or DES), late-lumen loss was comparable in either SCB/BCB or PCB with no significant difference in subgroups (p = 0.4 Fig. 6C).

**Fig. 7:**
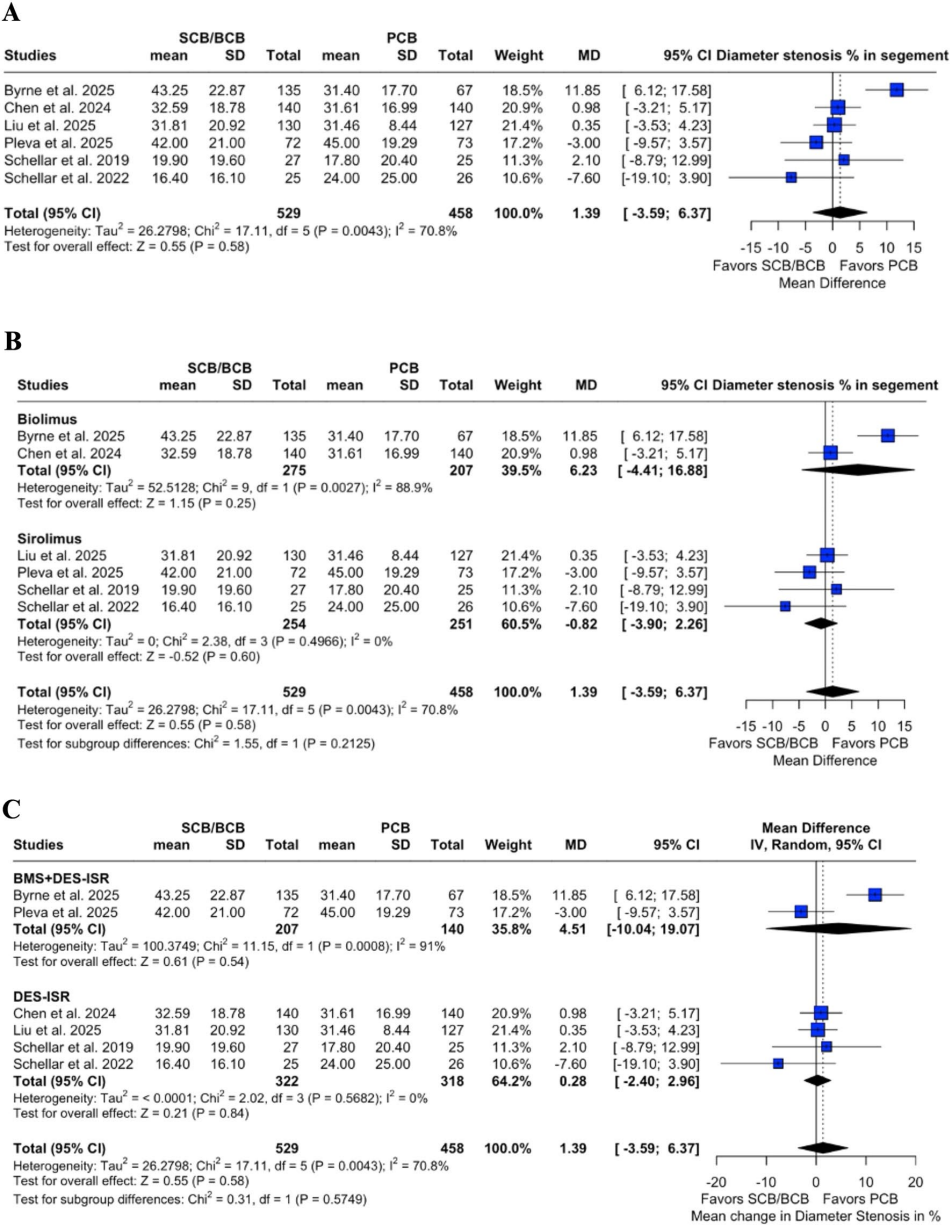
**A**: Pooled analysis of Diameter stenosis in % at follow-up comparing combined SCB and BCB with PCB in patients with Coronary ISR. **B**: Subgroup analysis of DS in % at follow-up comparing SCB or BCB with PCB in patients with Coronary ISR. **C**: Subgroup analysis based on type of prior stent (DES/BMS) In-stent restenosis (ISR) for DS in % at follow-up

**Fig. 8:**
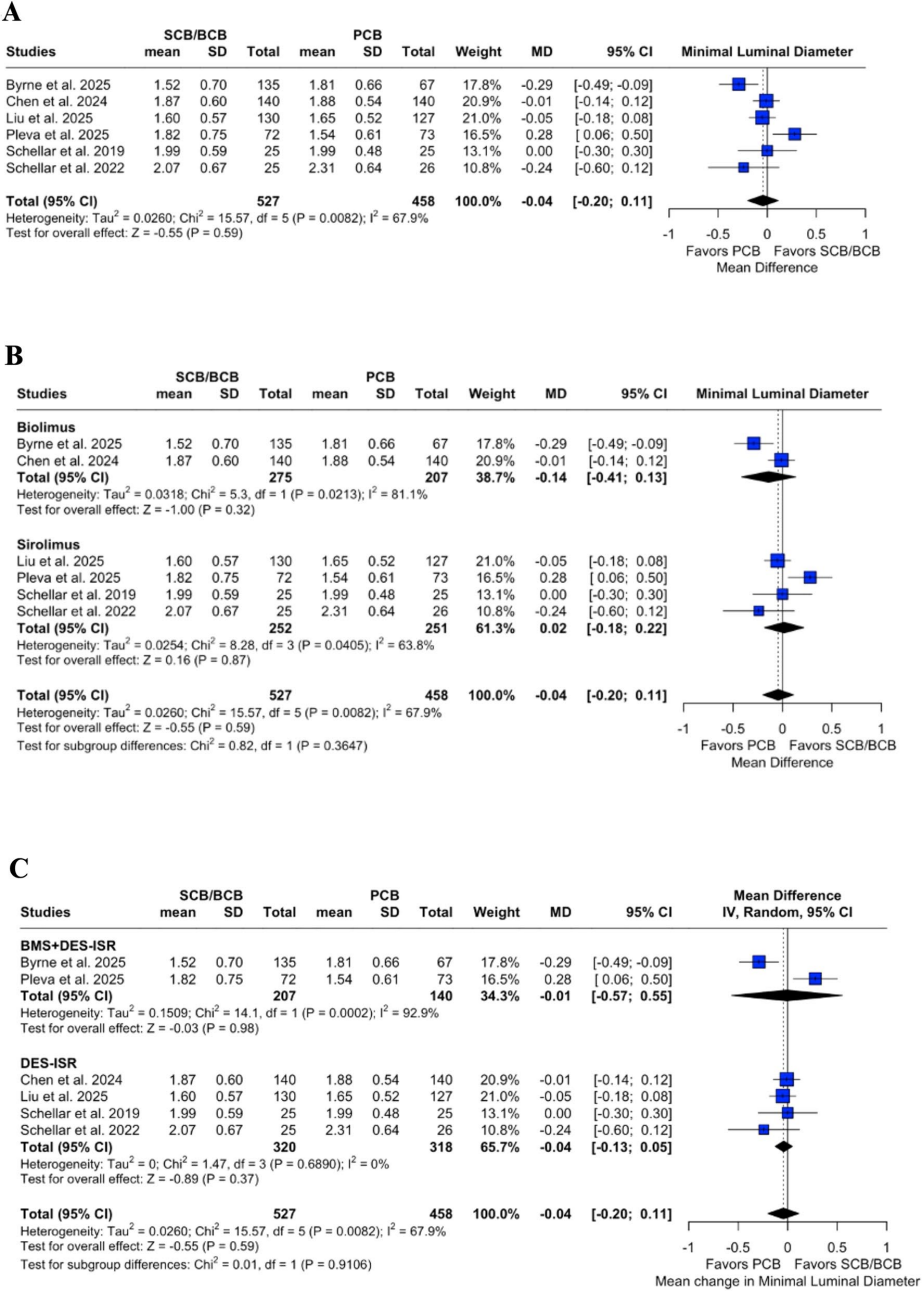
**A**: Pooled analysis of MLD at follow-up comparing combined SCB and BCB with PCB in patients with Coronary ISR. **B**: Subgroup analysis of MLD at follow-up comparing SCB or BCB with PCB in patients with Coronary ISR. **C**: Subgroup analysis based on type of prior stent (DES/BMS) In-stent restenosis (ISR) for MLD at follow-up

Diameter stenosis in percentage showed a mean difference of 1.39% (95% CI = -3.59, 6.37, p = 0.58, I^2^ = 71% Fig. 7A) between the two groups**.** When analyzed based on the type of balloon utilized, SCB showed MD of -0.82% with null heterogeneity** (**95% CI = -3.90, 2.26, p = 0.60, I^2^ = 0%), and BCB showed MD of 6.23% (95% CI = -4.41, 16.88, p = 0.25, I^2^ = 89%) **(**Fig. 7B)*.* Subgroup analysis based on type of stent of ISR, also showed similar results (Fig. 7C). Minimal Lumen diameter showed a reduction of 0.04 mm (95% CI: -0.20, 0.11, p = 0.59, I^2^ = 68%) with SCB/BCB group vs PCB group (Fig. 8A), when analyzed based on the type of balloon used, SCB and BCB independently showed no significant difference compared to PCB (Fig. 8B). Similar results were also noted when analyzing subgroups based on type of stent of ISR (Fig. 8C).

**Fig. 9:**
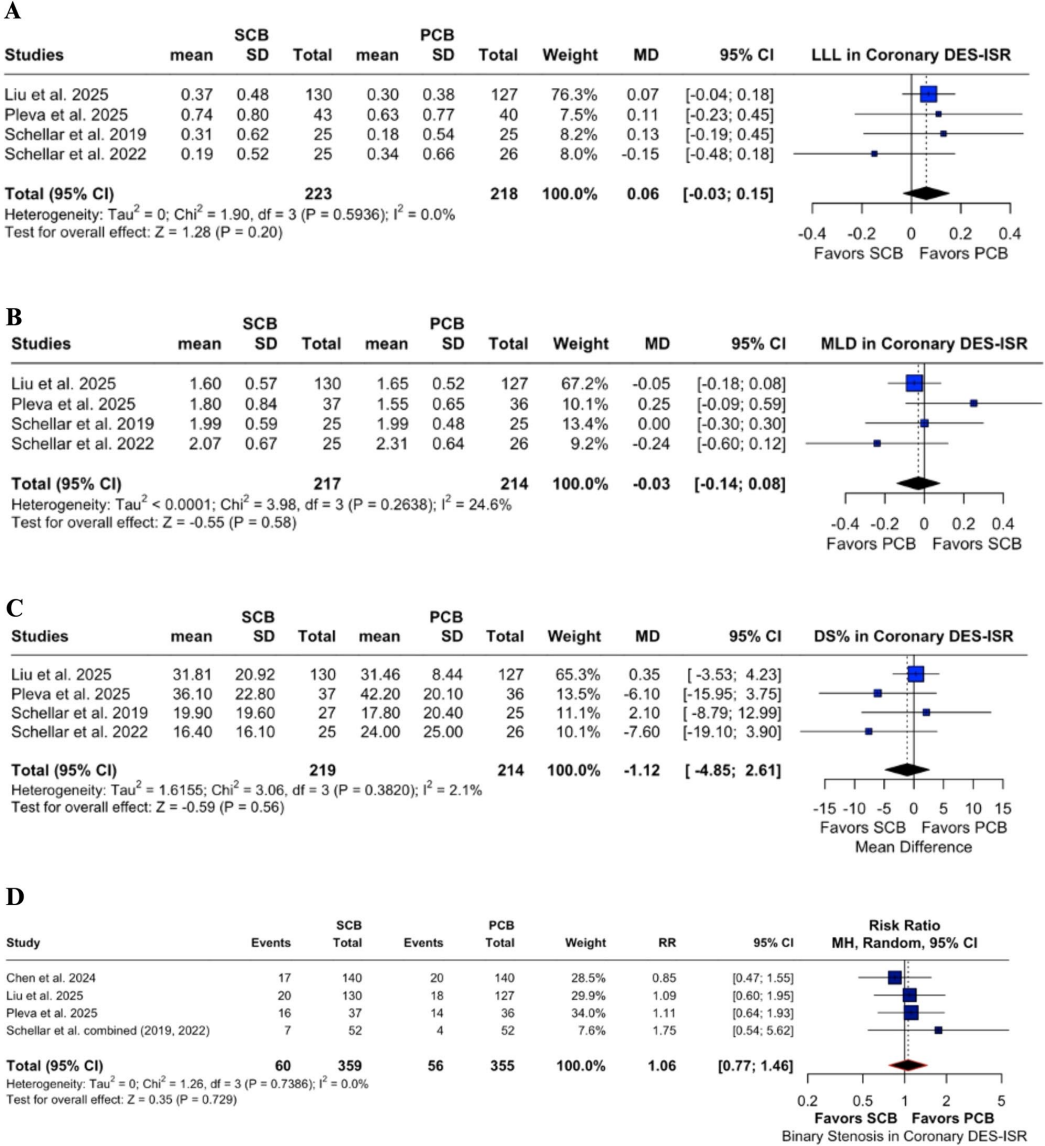
**A**: LLL at follow-up comparing SCB and PCB in patients with Coronary DES-ISR. **B**: MLD at follow-up comparing SCB and PCB in patients with Coronary DES-ISR. **C**: Diameter stenosis in % at follow-up comparing SCB and PCB in patients with Coronary DES-ISR. **D**: BS in segment at follow-up comparing SCB and PCB in patients with Coronary DES-ISR. SCB: Sirolimus-coated balloons, BCB: Biolimus-coated balloons, PCB: Paclitaxel-coated balloons, RR: relative risk, CI: confidence interval, M-H: Mantel-Haenzel, MD: mean difference, SD: Standard deviation, DES: Drug Eluting Stent, ISR: In-stent restenosis, BMS: Bare Metal Stent

When analyzing selectively SCB vs PCB in patients with coronary DES-ISR, late lumen loss, minimal lumen diameter, diameter stenosis in % and binary stenosis in segment all showed no statistically significant difference between the two groups (Fig. 9A ,9B, 9C, 9D).

The absence of statistical significance in this study’s outcomes could be due to inadequate sample size, short follow-ups or a smaller number of events.

Subgroup analysis based on individual use of either SCB or BCB compared to PCB and type of stent of ISR, either DES + BMS or DES, for the outcomes of Target lesion revascularization, all-cause mortality, target vessel myocardial infarction, binary restenosis, late lumen loss, diameter stenosis in % and minimal lumen diameter also exhibited similar results as the overall pooled analysis.

### Sensitivity analysis

Heterogeneity is null for most of the outcomes, suggesting consistency across the included studies. However, few outcomes, like late lumen loss, diameter stenosis percentage, minimal lumen diameter, and binary restenosis, revealed high heterogeneity, which was assessed by leave-one-out analysis (Supplementary Figs. 9.1—9.4). Sensitivity analysis was conducted by omitting one study at a time via leave-one-out analysis, which significantly eliminated the heterogeneity and maintained the direction of association, further indicating robust results. With the low level of heterogeneity and the drop in heterogeneity upon leave-one-out sensitivity analysis, there is a level of consistency and uniformity across the included studies.

Funnel plots were also performed to assess publication bias of each outcome, where the horizontal axis represents effect size, either MD or RR, and the vertical axis represents standard error (SE) of the effect size. Each circle represents an individual study, with the size indicating study precision—larger distances from the dashed line at zero may indicate potential bias or variability in study results. The symmetric appearance of the funnel plot generally indicates a low risk of publication bias. However, asymmetry may suggest selective publication or other biases impacting the results. The point farther from the cluster might indicate a study with distinct results, warranting further investigation. On examining all the funnel plots, mild asymmetry is noted in outcomes like binary restenosis in the segment, LLL, diameter stenosis in percent, and MLD *(supplementary Figs. 10.4 to 10.7),* indicating consistency of the outcome in all studies and minimal bias. Funnel plot of TLR and Target vessel MI *(supplementary Fig. 10.1 and 10.3)* shows relative symmetry with one study appearing as an outlier, potentially due to different methodologies or populations, indicating the need for further exploration. Visual inspection shows moderate asymmetry with mortality outcome, suggesting some degree of publication bias or heterogeneity among the included studies, but not substantial, highlighting the need for cautious interpretation of pooled results *(supplementary Fig. 10.2).*

### Quality assessment

Risk of bias was evaluated by two independent authors (C.L. and J.M.) at the study level using the Cochrane Risk of Bias 2 (RoB 2) tool across the following domains: [1] bias arising from the randomization process; [2] bias due to deviations from intended interventions; [3] bias resulting from missing outcome data; [4] bias in outcome measurement; [5] bias in selection of the reported results; and [6] overall risk of bias. All included RCTs were judged to have a low risk of bias, as illustrated in *Supplemental Fig. 11*.

### GRADE assessment and certainty of evidence

The certainty of evidence for each outcome was assessed using the GRADE framework, and a summary of findings table was created *(supplementary Table 3).* Overall, the evidence ranged from moderate to very low certainty. For target lesion revascularization (TLR), the relative effect estimates favored neither SCB/BCB nor PCB definitively, with low certainty due to imprecision and risk of bias. Mortality and target vessel myocardial infarction were rare, resulting in wide confidence intervals and low certainty ratings. Binary restenosis showed high variability, particularly with BCBs, leading to very low certainty due to serious inconsistency and imprecision. Continuous outcomes, including late lumen loss, diameter stenosis percentage, and minimal lumen diameter, showed no statistically significant differences between treatment groups. These were generally rated as low certainty, except in the SCB vs PCB subgroup for late lumen loss, which reached moderate certainty due to consistent and precise estimates. The overall body of evidence suggests no clear superiority of SCB/BCB over PCB, with limitations in precision, consistency, and event frequency reducing confidence in the findings.

## Discussion

In this pooled analysis, SCB and BCB demonstrated comparable efficacy and safety outcomes to PCB across multiple clinical endpoints. Although SCB/BCB use was associated with a non-significant trend toward higher TLR rates (RR = 1.24), no clear superiority or inferiority of either strategy was evident. Similar non-significant differences were observed for mortality (p = 0.78), target vessel myocardial infarction (p = 0.43), binary restenosis (p = 0.24), late lumen loss (p = 0.31), diameter stenosis (p = 0.61), and minimal lumen diameter (p = 0.56). The absence of statistical significance across endpoints may reflect limitations such as small sample sizes, short follow-up durations, and low event rates, which could reduce the power to detect clinically meaningful differences. Despite stratified analysis based on type of DCB used and whether DES or BMS-ISR, no significant differences were found in all the outcomes.

A summary of the main types of drug-coated balloons, including their indications, mechanisms of action, and contraindications, is provided in Table 2 (*C*omparison of Drug-Coated Balloon Types for Coronary ISR) [20–23, 26–28]. ISR is defined as a luminal re-narrowing of greater than 50% within the stented segment or within 5 mm of its edges, as confirmed by coronary angiography or intravascular imaging modalities [15, 16]. This phenomenon reflects neointimal hyperplasia or neo-atherosclerosis occurring after PCI, and it typically presents clinically with recurrent ischemic symptoms such as angina or, less commonly, acute coronary syndromes [17]. The incidence of ISR varies according to stent type, lesion complexity, and follow-up duration. In the contemporary era of DES, randomized trials and large registries report ISR rates of approximately 5% to 10% within the first year [18, 19]. Long-term follow-up data from U.S. Medicare beneficiaries have demonstrated cumulative ISR rates ranging between 5 and 20% at five years post-PCI [20]. A recent meta-analysis pooling global data estimated a DES-ISR incidence of around 13% (95% confidence interval [CI] 10–15%) [21–23].

**Table 2:** Comparison of all the types of balloons compared

Drug-coated balloon type	Mechanism of action	Indications	Contraindications
Paclitaxel-coated balloon (PCB)	Paclitaxel binds to β-tubulin, stabilizing microtubules and inhibiting smooth muscle cell proliferation and migration. Rapid tissue uptake with sustained antiproliferative effect due to high lipophilicity	Treatment of coronary in-stent restenosis (BMS-ISR and DES-ISR); small vessel disease; avoidance of additional stent layers	Severe vessel calcification preventing adequate balloon expansion; acute vessel thrombosis; untreated dissection requiring scaffold; hypersensitivity to paclitaxel
Sirolimus-coated balloon (SCB)	Sirolimus binds FKBP-12, inhibiting the mTOR pathway, blocking cell cycle progression from G1 to S phase, and reducing smooth muscle proliferation and neointimal growth. Requires carrier technology for effective tissue transfer and sustained release	Treatment of coronary ISR (BMS-ISR and DES-ISR) where limus-based therapy is preferred; small vessel disease; lesions requiring a metal-free approach	Acute vessel thrombosis; underexpanded stent not corrected; hypersensitivity to sirolimus or excipients; lack of data in certain high-thrombosis-risk scenarios
Biolimus-coated balloon (BCB)	Biolimus is a semi-synthetic sirolimus analogue with higher lipophilicity, allowing deeper arterial wall penetration; it inhibits the mTOR pathway, suppressing smooth muscle cell proliferation and neointimal hyperplasia	Emerging option for DES-ISR; potential use in patients where limus-based antiproliferative therapy is indicated and avoidance of further stent layers is desired	Limited evidence base—use with caution in unstudied populations; hypersensitivity to biolimus; acute vessel thrombosis; underexpanded stent not corrected

Comparison of Drug-Coated Balloon Types for Coronary ISR. BMS: Bare-Metal Stent; ISR: In-Stent Restenosis; DES: Drug-Eluting Stent; PCB: Paclitaxel-Coated Balloon; SCB: Sirolimus-Coated Balloon; BCB: Biolimus-Coated Balloon; FKBP-12: FK506 Binding Protein 12; mTOR: Mechanistic Target of Rapamycin. [20–23, 26–28]

Certain high-risk lesion subsets demonstrate substantially higher restenosis rates. For example, in patients undergoing DES implantation in the unprotected left main coronary artery, angiographic restenosis rates range from 8 to 42% depending on lesion location and stenting technique [24]. Geographical differences have also been observed, with U.S. series reporting ISR incidences of approximately 27% (95% CI 20.6–33.9%), compared with about 13.6% in Asian cohorts [25]. These figures underscore that, despite significant advances in stent technology and adjunctive pharmacotherapy, ISR remains a relevant clinical challenge worldwide, particularly in complex anatomical settings and high-risk patient populations. Ongoing innovations in stent design, drug-delivery technologies, and vessel-healing modulation aim to further reduce its incidence and improve long-term vessel patency.

DCB is an established option for treating coronary ISR, particularly when avoiding additional metal layers is desirable. The best-validated class is the PCB, which has demonstrated consistent angiographic and clinical efficacy across randomized trials. In DES-ISR, ISAR-DESIRE 3 showed PCB reduced repeat revascularization versus plain balloon angioplasty and performed similarly to a paclitaxel-eluting stent, supporting a no-new-metal strategy with durable outcomes (angiographic late lumen loss and target lesion revascularization) [26]. In PEPCAD China ISR, PCB was non-inferior to paclitaxel-eluting stents for in-segment late lumen loss and clinical endpoints, reinforcing PCB as a standard comparator in ISR [27]. Although RIBS IV/V favored repeat everolimus-eluting stenting over PCB for some efficacy endpoints, both trials confirmed PCB as an active therapy, particularly attractive when adding another stent layer is undesirable [28, 29]. Longer-term follow-up and meta-analytic syntheses continue to support PCB effectiveness and safety in ISR populations [23, 30]. Representative trialed platforms include SeQuent Please (iopromide-based coating) and IN.PACT Falcon (urea-based) [31, 32].

Evidence for SCB has matured from early feasibility to randomized comparisons versus PCB in ISR. Contemporary randomized trials have shown non-inferiority of SCB to PCB for in-segment late lumen loss at 6–9 months, with similar early clinical outcomes, indicating class equipoise between limus- and paclitaxel-based balloons in ISR [33–35]. The multicenter SIBLINT-ISR randomized trial further corroborated that SCB achieves non-inferior late lumen loss to PCB at 9 months in ISR, with comparable short-term clinical events. Available SCB platforms in studies and contemporary practice include SeQuent SCB, MagicTouch, and Selution SLR, which pair sirolimus with novel carriers to enhance tissue bioavailability [28].

Data for BCB in ISR are emerging. Early randomized and prospective studies suggest biolimus-based balloons may achieve angiographic outcomes comparable to PCB in DES-ISR, but sample sizes and follow-up are more limited; thus, BCBs are promising yet less extensively validated than PCB and SCB [28, 35]. Across DCB classes, mechanism-guided selection with IVUS/OCT is recommended: DCB is particularly attractive for layered ISR, small vessel disease, or when prior under expansion has been corrected and additional scaffolding is undesirable; repeat stenting is generally reserved for scenarios requiring a new scaffold (stent fracture or edge-related mechanical failure).

When contextualized with the existing literature, our pooled analysis reinforces and extends current evidence on the relative performance of SCB/BCB compared with PCB for ISR. Consistent with prior randomized trials and meta-analyses, which have established PCB as the most extensively validated DCB technology with robust angiographic and clinical efficacy in ISR [13, 26, 27], we found no statistically significant differences in key endpoints, including TLR, mortality, target vessel myocardial infarction, binary restenosis, late lumen loss, diameter stenosis, and minimal lumen diameter, between SCB/BCB and PCB. This aligns with recent randomized data, such as SIBLINT-ISR [2] and other non-inferiority trials which have demonstrated comparable angiographic outcomes and early clinical event rates between SCB and PCB, as well as emerging prospective evidence suggesting that BCB achieves similar efficacy in DES-ISR despite more limited validation. Notably, our observed non-significant trend toward higher TLR with SCB/BCB (RR = 1.24) is consistent with the variability seen in individual trial point estimates and may reflect the smaller aggregate sample sizes, shorter follow-up, and low event rates characteristic of the available evidence base. Taken together, both our findings and prior studies support a position of therapeutic equivalence among current DCB classes for ISR, though device selection criteria could not be derived as studies included has comparable baseline characteristics and subgroup analysis did not reveal any difference in results.

## Study limitations

This study has several limitations. First, considerable heterogeneity was observed across the included RCTs concerning binary in-segment restenosis, LLL, percentage diameter stenosis within the segment, and in-segment MLD. This variability may be attributable to differences in the types of SCB and BCB employed in the individual trials, as device characteristics can influence procedural efficacy and angiographic outcomes. There are only two studies that compared BCB vs PCB, although both the studies have modest population the number is comparable with pooled SCB group.

Second, this meta-analysis included studies irrespective of the type of PCB or SCB/BCB utilized. Given the absence of a proven class effect among DCBs, pooling data from trials using different devices may have introduced additional study-level heterogeneity, as previously discussed. Among the two studies comparing BCB with PCB contrasting outcomes were observed. The REFORM study failed to demonstrate non-inferiority of BCB compared to PCB in angiographic outcomes, though clinical outcomes were comparable at 1 year follow-up. Conversely, BIO-ASCEND ISR study found similar outcomes between both the treatment groups. Given that both the studies are underpowered with modest sample sizes, we conducted a pooled analysis to investigate whether combined results might reveal differential outcomes not apparent in individual studies.

Third, the maximum duration of clinical follow-up across the included RCTs was 12 months. Consequently, this analysis could not evaluate the long-term safety and efficacy of DCB-PCI, including the durability of anti-restenotic effects or potential late adverse events.

Fourth, and a significant mechanistic limitation, none of the included trials incorporated routine intravascular imaging modalities such as intravascular ultrasound (IVUS) or optical coherence tomography (OCT) for follow-up. The absence of these advanced imaging techniques precludes a detailed understanding of the mechanistic insights into vessel healing patterns, precise neointimal response, and micro-structural changes after DCB application [36–38]. IVUS and OCT are invaluable for assessing aspects such as the extent of neointimal hyperplasia, the degree of stent strut coverage, and the presence of residual dissections or mal-apposition – factors crucial for explaining long-term clinical outcomes [37]. Without such detailed imaging, it is challenging to fully elucidate the biological effects and differential performance of various DCB types at a sub-clinical level, limiting our ability to pinpoint the exact reasons for observed clinical outcomes or lack thereof.

Finally, the total sample size across the included trials remains relatively modest, which may limit the statistical power to detect more minor but clinically relevant differences in outcomes and only two studies compared BCB vs PCB but the total population of BCB group and SCB group were comparable.

A notable observation in our meta-analysis was the very high heterogeneity (I^2^ > 80–90%) observed in the analyses of late lumen loss (LLL) and diameter stenosis, particularly for the BCB group. This substantial heterogeneity is unlikely to be solely explained by limited sample size and warrants a more in-depth consideration of potential clinical and technical contributing factors. Variations across studies in BCB platforms and their underlying drug-delivery carrier technologies likely play a significant role. Different biolimus formulations, excipients, and coating designs can influence drug release kinetics, tissue penetration, and local vessel wall response, leading to diverse angiographic outcomes [34]. Furthermore, differences in the trial design characteristics, such as patient selection (e.g., lesion complexity, vessel anatomy), procedural details (e.g., extent of pre-dilatation, balloon inflation duration and pressure), and angiographic assessment protocols could also contribute to this variability. These subtle but important distinctions between devices and procedural approaches underscore the challenges in directly comparing data from studies utilizing different technologies and highlight the need for further research to standardize DCB platforms and procedural techniques to minimize outcome variability.

## Conclusion

In conclusion our meta-analysis demonstrated that m-TOR inhibitor i.e. Limus coated Balloons particularly SCB are non-inferior to PCB for the treatment of coronary ISR across multiple angiographic and clinical endpoints, and this was consistent even upon stratification by type of ISR (DES or BMS) or specific DCB type (SCB/BCB). However, the interpretation of these findings requires caution as the available evidence for several outcomes was rated as low to very low certainty according to GRADE, reflecting limitations such as a restricted number of direct randomized controlled trials (particularly for BCBs) and heterogeneity in some endpoints. Therefore, while our findings suggest similar clinical performance between these DCB platforms, a definitive declaration remains unclear by the current body of evidence, highlighting the need for larger, high-quality randomized controlled trials with longer-term follow-up.

## Supplementary Information

Below is the link to the electronic supplementary material.

Supplementary file 1.

## Data Availability

The data used in this meta-analysis were extracted from publicly available sources, including published randomized controlled trials. All relevant data supporting the findings of this study are included within the manuscript and supplementary materials. Due to the nature of this analysis, no new primary data were generated. Additional details can be made available upon reasonable request to the corresponding author.
