# Supplementary material for: Drug-coated balloons in coronary in-stent restenosis: a systematic review and meta-analysis comparing sirolimus and biolimus with paclitaxel platforms
Source: Cardiovasc Interv Ther. 2026 Mar 3;41(3):491–509. doi: 10.1007/s12928-026-01248-4 (PMC13279376; doi:10.1007/s12928-026-01248-4)

**SUPPLEMENTARY APPENDIX**

**Sirolimus/Biolimus vs Paclitaxel Coated Balloons in Coronary in-stent Restenosis: A Systematic Review and Meta-Analysis**

| Population | Patients with CAD, H/o PCI with coronary in-stent restenosis |
| --- | --- |
| Intervention | Sirolimus/Biolimus-coated balloon |
| Control | Paclitaxel-coated Balloon |
| Outcome | P: ischemic events, myocardial infarction (MI), and all-cause death  S: Periprocedural complications, Binary restenosis target vessel revascularization (TVR), stent thrombosis (ST), major bleeding |
| Type of Study | RCT |
| Time of follow-up | No restrictions |

Inclusion Criteria:

- ≥18 years of age with CAD with prior PCI, presenting with in-stent restenosis
- Direct comparison between sirolimus or biolimus vs paclitaxel-coated stents
- RCTs, with either subgroups or the main study
- Reported at least one of the outcomes of interest

Exclusion Criteria:

- Acute MI
- New PCI, No prior intervention
- Acute CHF/Cardiogenic shock
- Single-arm studies with no control
- Case reports/ case series/ conference abstracts
- Studies that were not published

Search Formulae:

("Sirolimus-Coated Balloon*" OR "SCB" OR "Sirolimus-Eluting Balloon*" OR "Rapamycin-Coated Balloon*" OR "Sirolimus DCB" OR sirolimus OR rapamycin OR "Everolimus coated balloon” OR “everolimus” OR "biolimus" OR “limus coated balloon” OR "mTOR inhibitors") AND ("Paclitaxel-Coated Balloon*" OR "PCB" OR "Paclitaxel-Eluting Balloon*" OR "Paclitaxel DCB" OR paclitaxel) AND (balloon* OR "drug-coated balloon*" OR "drug-eluting balloon*") AND ("Coronary In-Stent Restenosis" OR "In-Stent Restenosis" OR "ISR" OR "Coronary Restenosis" OR "Stent Restenosis" OR restenosis OR "Coronary stenosis")

| **Section and Topic** | **Item #** | **Checklist item** | **Location where item is reported** |
| --- | --- | --- | --- |
| **TITLE** | | |  |
| Title | 1 | Identify the report as a systematic review. | Page 1 |
| **ABSTRACT** | | |  |
| Abstract | 2 | See the PRISMA 2020 for Abstracts checklist. | Page 3 |
| **INTRODUCTION** | | |  |
| Rationale | 3 | Describe the rationale for the review in the context of existing knowledge. | Page 4-5 |
| Objectives | 4 | Provide an explicit statement of the objective(s) or question(s) the review addresses. | Page 5 |
| **METHODS** | | |  |
| Eligibility criteria | 5 | Specify the inclusion and exclusion criteria for the review and how studies were grouped for the syntheses. | Page 5 |
| Information sources | 6 | Specify all databases, registers, websites, organisations, reference lists and other sources searched or consulted to identify studies. Specify the date when each source was last searched or consulted. | Page 6 |
| Search strategy | 7 | Present the full search strategies for all databases, registers and websites, including any filters and limits used. | Page 6 |
| Selection process | 8 | Specify the methods used to decide whether a study met the inclusion criteria of the review, including how many reviewers screened each record and each report retrieved, whether they worked independently, and if applicable, details of automation tools used in the process. | Page 6 |
| Data collection process | 9 | Specify the methods used to collect data from reports, including how many reviewers collected data from each report, whether they worked independently, any processes for obtaining or confirming data from study investigators, and if applicable, details of automation tools used in the process. | Page 6 |
| Data items | 10a | List and define all outcomes for which data were sought. Specify whether all results that were compatible with each outcome domain in each study were sought (e.g. for all measures, time points, analyses), and if not, the methods used to decide which results to collect. | Page 6 |
|  | 10b | List and define all other variables for which data were sought (e.g. participant and intervention characteristics, funding sources). Describe any assumptions made about any missing or unclear information. | Page 6, Table 1 |
| Study risk of bias assessment | 11 | Specify the methods used to assess risk of bias in the included studies, including details of the tool(s) used, how many reviewers assessed each study and whether they worked independently, and if applicable, details of automation tools used in the process. | Page 7 |
| Effect measures | 12 | Specify for each outcome the effect measure(s) (e.g. risk ratio, mean difference) used in the synthesis or presentation of results. | Page 7 |
| Synthesis methods | 13a | Describe the processes used to decide which studies were eligible for each synthesis (e.g. tabulating the study intervention characteristics and comparing against the planned groups for each synthesis (item #5)). | Page 7 |
|  | 13b | Describe any methods required to prepare the data for presentation or synthesis, such as handling of missing summary statistics, or data conversions. | Page 7 |
|  | 13c | Describe any methods used to tabulate or visually display results of individual studies and syntheses. | Page 7-10 |
|  | 13d | Describe any methods used to synthesize results and provide a rationale for the choice(s). If meta-analysis was performed, describe the model(s), method(s) to identify the presence and extent of statistical heterogeneity, and software package(s) used. | Page 6-7 |
|  | 13e | Describe any methods used to explore possible causes of heterogeneity among study results (e.g. subgroup analysis, meta-regression). | Page 8 |
|  | 13f | Describe any sensitivity analyses conducted to assess robustness of the synthesized results. | Page 9 |
| Reporting bias assessment | 14 | Describe any methods used to assess risk of bias due to missing results in a synthesis (arising from reporting biases). | Page 10 |
| Certainty assessment | 15 | Describe any methods used to assess certainty (or confidence) in the body of evidence for an outcome. | Page 10,  Figure 13 |
| **RESULTS** | | |  |
| Study selection | 16a | Describe the results of the search and selection process, from the number of records identified in the search to the number of studies included in the review, ideally using a flow diagram. | Page 7-8, Figure 2 |
|  | 16b | Cite studies that might appear to meet the inclusion criteria, but which were excluded, and explain why they were excluded. | Figure 2 |
| Study characteristics | 17 | Cite each included study and present its characteristics. | Table 1 |
| Risk of bias in studies | 18 | Present assessments of risk of bias for each included study. | Figure 13 |
| Results of individual studies | 19 | For all outcomes, present, for each study: (a) summary statistics for each group (where appropriate) and (b) an effect estimate and its precision (e.g. confidence/credible interval), ideally using structured tables or plots. | Page 7-8 |
| Results of syntheses | 20a | For each synthesis, briefly summarise the characteristics and risk of bias among contributing studies. | Page 7-10 |
|  | 20b | Present results of all statistical syntheses conducted. If meta-analysis was done, present for each the summary estimate and its precision (e.g. confidence/credible interval) and measures of statistical heterogeneity. If comparing groups, describe the direction of the effect. | Page 7-10 |
|  | 20c | Present results of all investigations of possible causes of heterogeneity among study results. | Page 10 |
|  | 20d | Present results of all sensitivity analyses conducted to assess the robustness of the synthesized results. | Page 9 |
| Reporting biases | 21 | Present assessments of risk of bias due to missing results (arising from reporting biases) for each synthesis assessed. | Page 10 |
| Certainty of evidence | 22 | Present assessments of certainty (or confidence) in the body of evidence for each outcome assessed. | Page 10 |
| **DISCUSSION** | | |  |
| Discussion | 23a | Provide a general interpretation of the results in the context of other evidence. | Page 10-11 |
|  | 23b | Discuss any limitations of the evidence included in the review. | Page 14-15 |
|  | 23c | Discuss any limitations of the review processes used. | Page 14-15 |
|  | 23d | Discuss implications of the results for practice, policy, and future research. | Page 16 |
| **OTHER INFORMATION** | | |  |
| Registration and protocol | 24a | Provide registration information for the review, including register name and registration number, or state that the review was not registered. | Page 7 |
|  | 24b | Indicate where the review protocol can be accessed, or state that a protocol was not prepared. | Page 7 |
|  | 24c | Describe and explain any amendments to information provided at registration or in the protocol. | Page 7 |
| Support | 25 | Describe sources of financial or non-financial support for the review, and the role of the funders or sponsors in the review. | Page 16 |
| Competing interests | 26 | Declare any competing interests of review authors. | Page 16 |
| Availability of data, code and other materials | 27 | Report which of the following are publicly available and where they can be found: template data collection forms; data extracted from included studies; data used for all analyses; analytic code; any other materials used in the review. | Page 17 |

Table 3: Summary of Findings (GRADE assessment)

| Outcome | No. of participants (studies) | Certainty of the evidence (GRADE) | Relative effect (95% CI) | Anticipated absolute effects | Risk with PCB | Risk difference with SCB/BCB |
| --- | --- | --- | --- | --- | --- | --- |
| Target lesion revascularization (SCB/BCB vs PCB) | 985 (RCTs:6) | Low | RR 1.24 (0.91–1.69) | 278 vs 167 per 1000 | 167 per 1000 | 111 per 1000 |
| Target lesion revascularization (SCB vs PCB) | 503 (RCTs:4) | Low | RR 1.09 (0.73–1.63) | 147 vs 167 per 1000 | 167 per 1000 | -20 per 1000 |
| Target lesion revascularization (BCB vs PCB) | 482 (RCTs:2) | Low | RR 1.50 (0.92–2.43) | 101 vs 167 per 1000 | 167 per 1000 | -66 per 1000 |
| Mortality (SCB/BCB vs PCB) | 985 (RCTs:5) | Low | RR 0.87 (0.32–2.38) | 13 vs 19 per 1000 | 19 per 1000 | -6 per 1000 |
| Target vessel MI (SCB/BCB vs PCB) | 985 (RCTs:5) | Low | RR 0.71 (0.29–1.69) | 20 vs 17 per 1000 | 17 per 1000 | 3 per 1000 |
| Binary restenosis (SCB/BCB vs PCB) | 988 (RCTs:5) | Low | RR 1.30 (0.84–2.02) | 157 vs 208 per 1000 | 208 per 1000 | -51 per 1000 |
| Binary restenosis (SCB vs PCB) | 481 (RCTs:3) | Low | RR 1.15 (0.82–1.62) | 188 vs 220 per 1000 | 220 per 1000 | -32 per 1000 |
| Binary restenosis (BCB vs PCB) | 482 (RCTs:2) | Very Low | RR 1.65 (0.42–6.56) | 126 vs 211 per 1000 | 211 per 1000 | -85 per 1000 |
| Late lumen loss (SCB/BCB vs PCB) | 985 (RCTs:6) | Low | MD 0.07 mm (−0.06 to 0.20) | Not significant | - per 1000 | - |
| Late lumen loss (SCB vs PCB) | 503 (RCTs:4) | Moderate | MD 0.05 mm (−0.04 to 0.14) | Not significant | - per 1000 | - |
| Late lumen loss (BCB vs PCB) | 482 (RCTs:2) | Very Low | MD 0.15 mm (−0.20 to 0.51) | Not significant | - per 1000 | - |
| Diameter stenosis % (SCB/BCB vs PCB) | 985 (RCTs:6) | Low | MD 1.25% (−3.54 to 6.04) | Not significant | - per 1000 | - |
| MLD in segment (SCB/BCB vs PCB) | 985 (RCTs:6) | Low | MD −0.04 mm (−0.19 to 0.10) | Not significant | - per 1000 | - |

Summary of findings table generated according to GRADE methodology comparing Sirolimus-coated balloons (SCB) and Biolimus-coated balloons (BCB) versus Paclitaxel-coated balloons (PCB) for treatment of coronary in-stent restenosis.

Outcomes include dichotomous and continuous endpoints, with certainty ratings and explanations for downgrading across the GRADE domains (risk of bias, inconsistency, indirectness, imprecision, publication bias).

**Leave one out analysis:**

**Figure 9.1 Mean difference of Late lumen loss omitting each study to test for high heterogeneity**


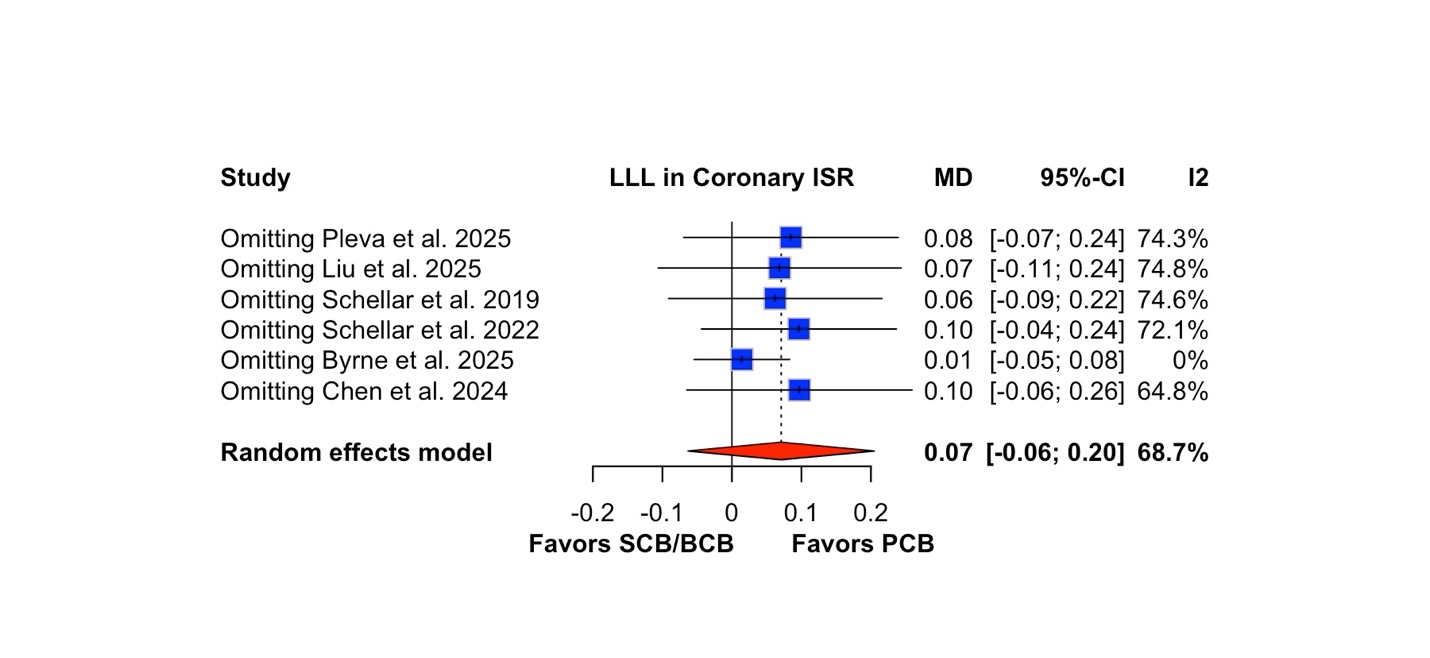


**Figure 9.2 Omitting each study for the outcome : diameter stenosis% for sensitivity analysis**


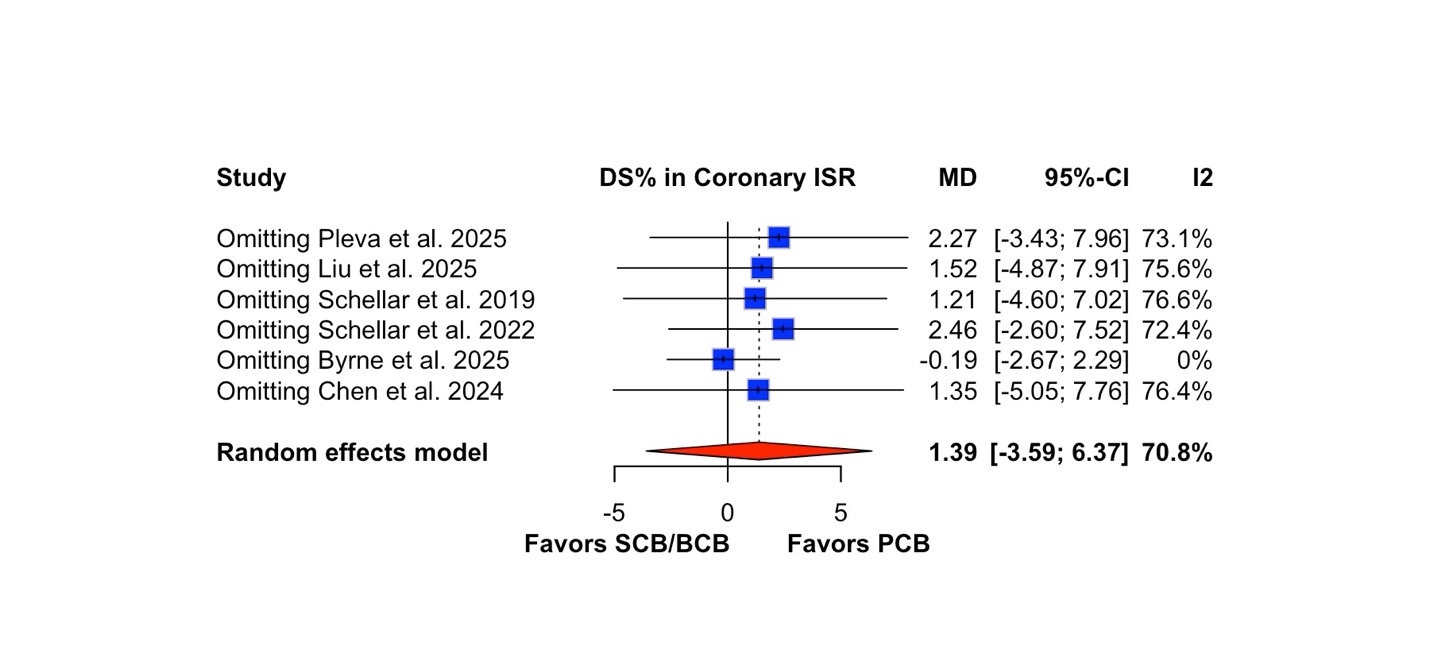


**Figure 9.3 Leave one out analysis of outcome Minimal lumen diameter omitting each study**

**
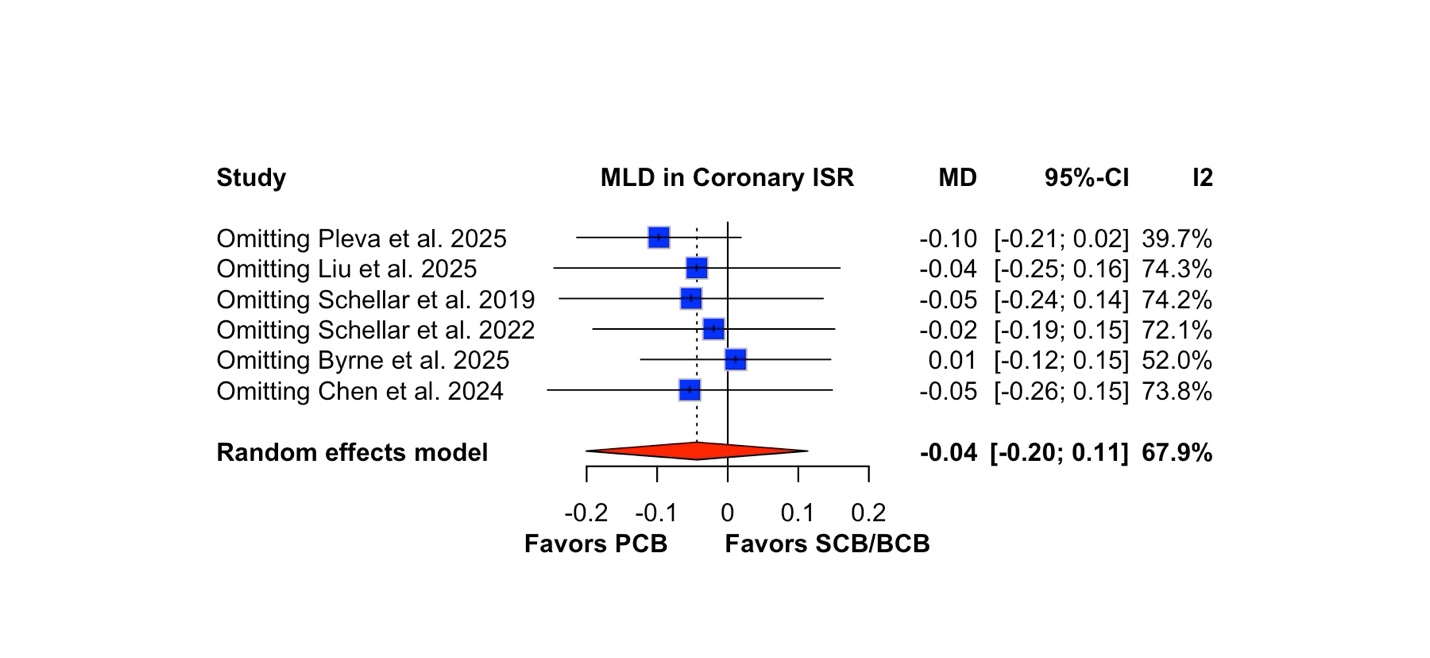
**

**Figure 9.4 Leave one out analysis of outcome : Binary restenosis in segment**


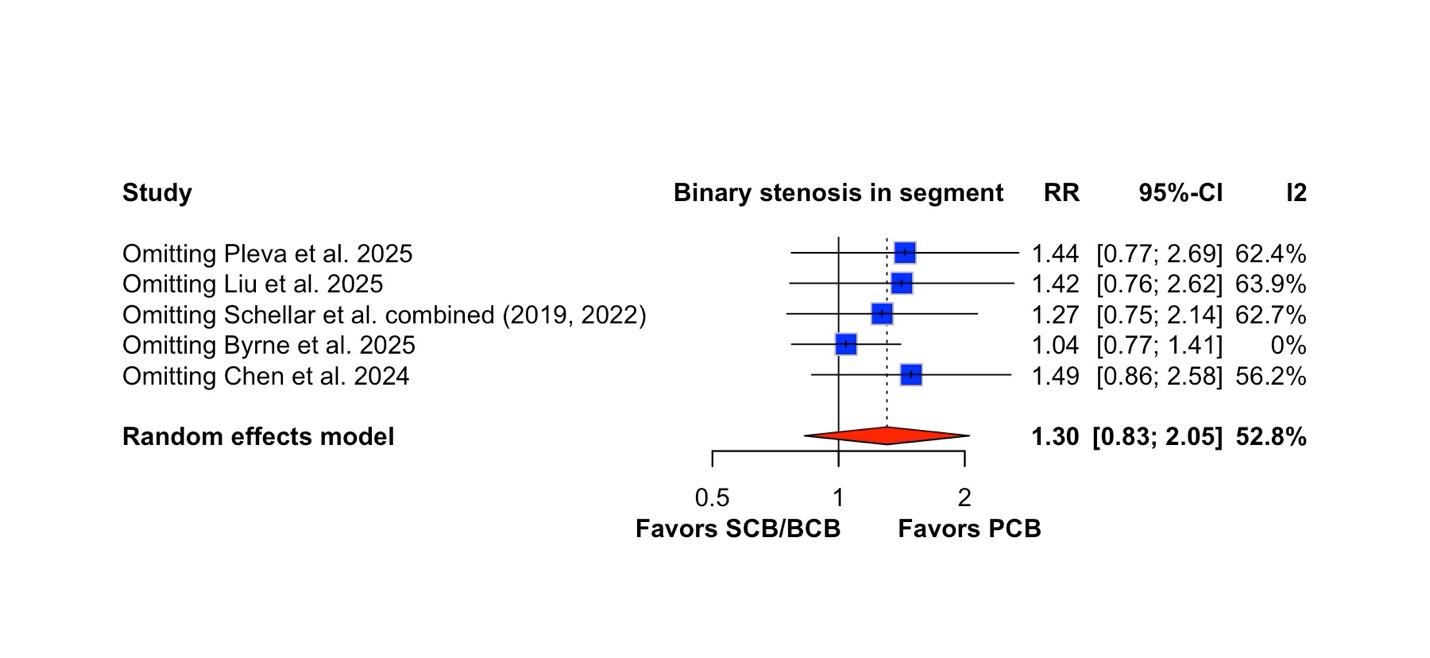


**Funnel plot of each outcome to assess publication Bias:**

**Figure 10.1 Symmetrical funnel plot of Target vessel revascularization showing no significant publication bias**


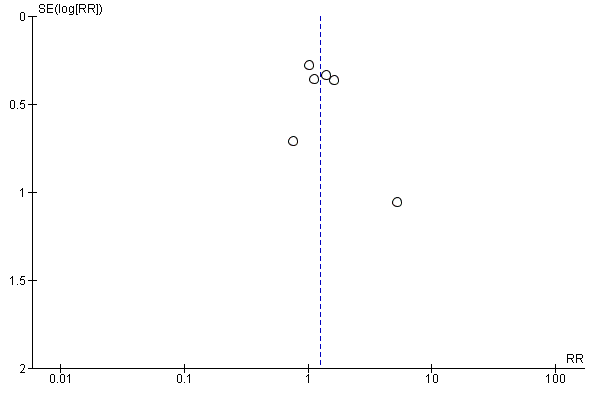


**Figure 10.2 Funnel plot for Mortality relatively symmetrical, suggesting minimal publication bias.**


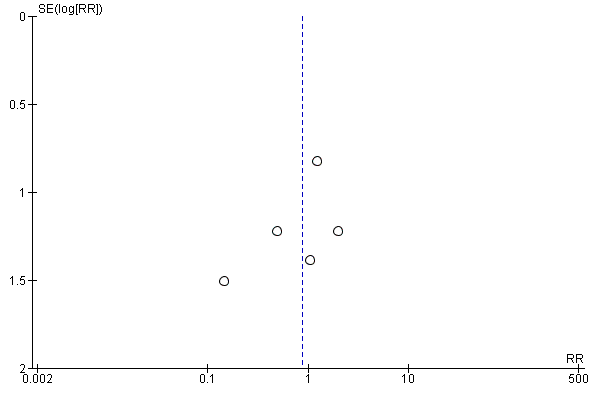


**Figure 10.3 Relatively symmetrical funnel plot of Target vessel MI showing minimal publication bias**


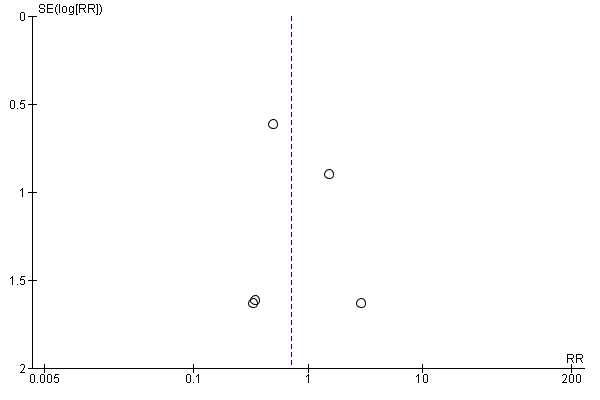


**Figure 10.4 Funnel plot of Binary restenosis in segment, showing symmetry indicating no significant publication bias**


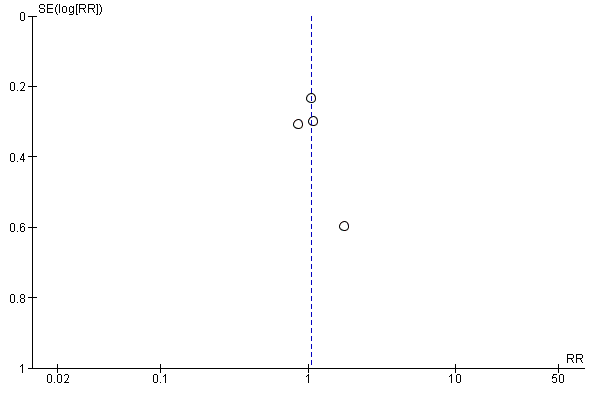


**Figure 10.5 Relatively symmetrical funnel plot of Late lumen loss, showing no significant publication bias**


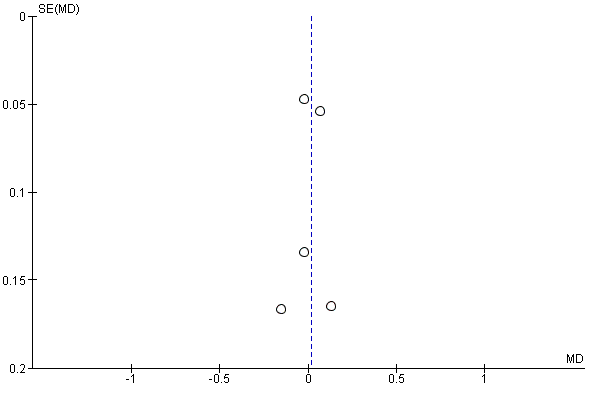


**Figure 10.6 Relatively symmetrical funnel plot of Diameter stenosis in % showing no significant publication bias**


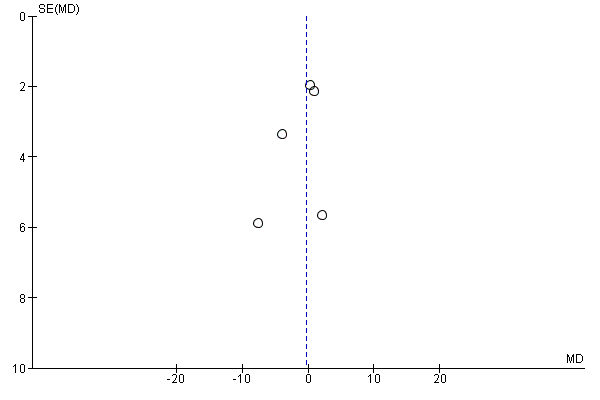


**Figure 10.7 Symmetrical funnel plot of MLD, showing no significant publication bias**


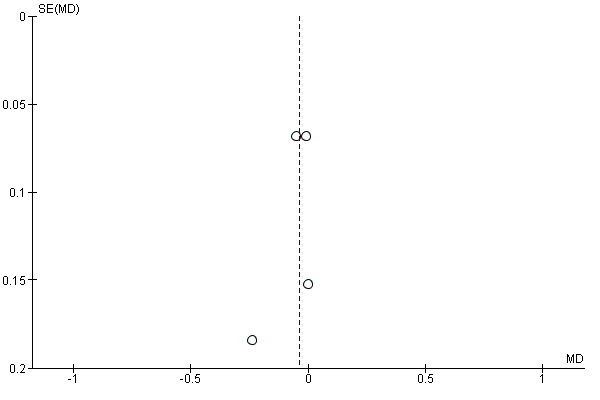


**Figure 11: Risk of Bias of all the studies assessing 5 domains**


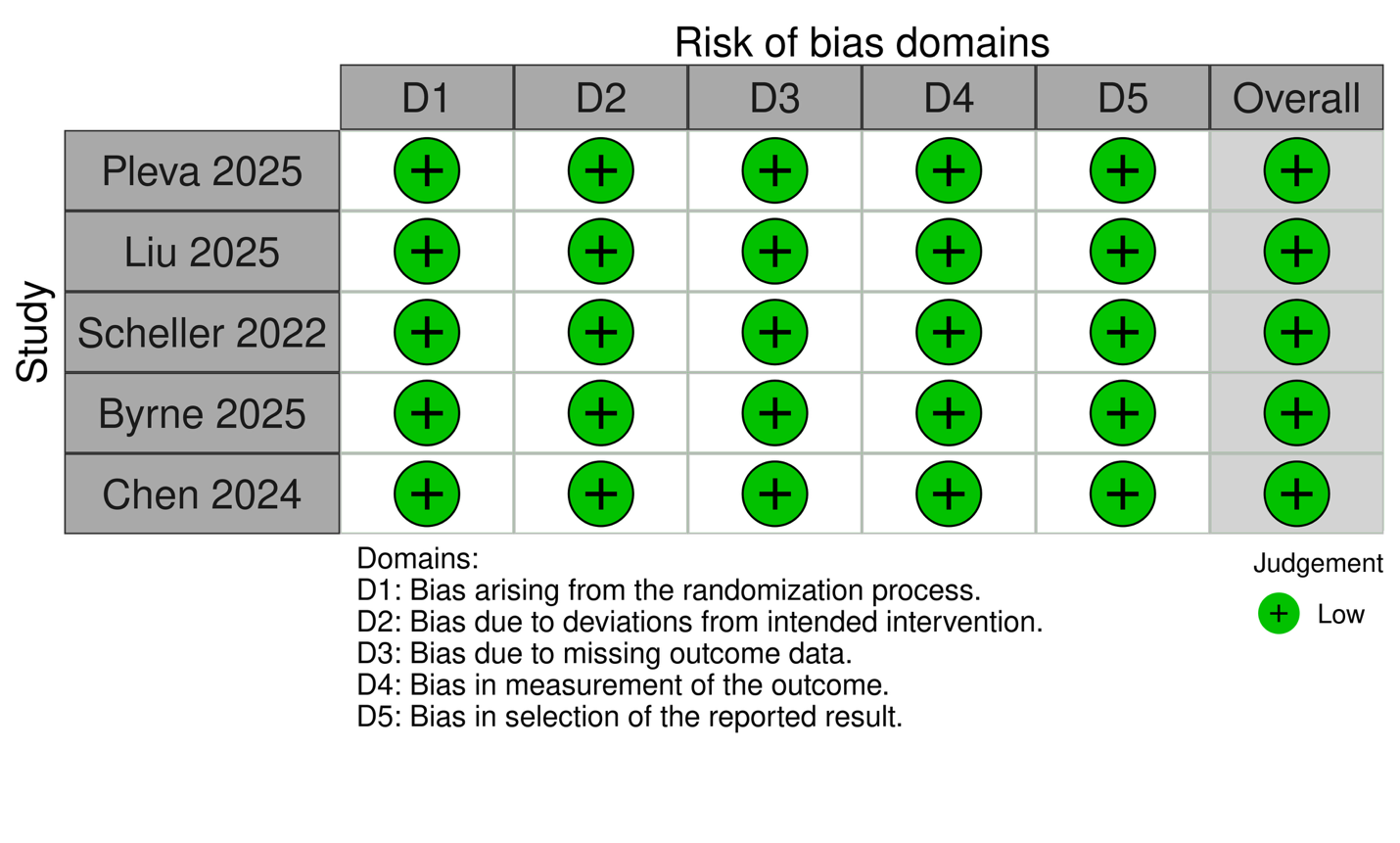

Supplement: Supplementary file 1 [file 12928_2026_1248_MOESM1_ESM.docx]
